# Erythrocyte Fraction in Thrombi Is Increased with Serum Iron by Influencing Fibrin Networks via Oxidative Stress

**DOI:** 10.1155/2021/3673313

**Published:** 2021-12-24

**Authors:** Mingli Liu, Minghui Chen, Zhongfei Hao, Qingbin Li, Yan Feng, Yongli Li, Ruiyan Li

**Affiliations:** The Department of Neurosurgery, Second Affiliated Hospital of Harbin Medical University, Harbin Medical University, Harbin, Heilongjiang, China

## Abstract

**Objective:**

This study investigated whether the erythrocyte fraction in thrombi would be increased with serum iron via oxidative stress.

**Methods:**

This study retrospectively enrolled patients with acute ischemic stroke treated using endovascular treatment in a single stroke center from October to December 2019. We examined the relationship between serum iron and erythrocyte-rich thrombi and the correlation of serum iron and the erythrocyte fraction in thrombi using clinical samples. Experiments *in vivo* and *in vitro* were performed to investigate the influence of oxidative stress on the correlation between serum iron concentration and erythrocyte fraction in thrombi.

**Results:**

We found from the clinical samples that serum iron concentration was related to erythrocyte-rich thrombi and positively associated with the erythrocyte fraction in thrombi *in vivo*. Further, the tightness of the fibrin networks regulating the erythrocyte fraction in thrombi was increased with serum iron concentration *in vivo*. Additionally, the oxidative stress level was increased with serum iron concentration *in vivo*. Moreover, we found that the tightness of the fibrin networks increased with higher oxidative stress levels *in vitro*. Lastly, experiments *in vivo* with inhibiting oxidative stress showed that the erythrocyte fraction in thrombi and the tightness of fibrin networks significantly increased in the iron group than those in the iron with oxidative stress inhibitor group and control group.

**Conclusions:**

Oxidative stress played a role in the process that the erythrocyte fraction in thrombi was increased with serum iron by influencing fibrin networks.

## 1. Introduction

Serum iron concentration reflects the overall iron status in the body and is associated with thrombosis [1, 2]. Iron-induced vascular dysfunction is known to contribute to the incidence of ischemic cardiovascular events by increasing vascular oxidative stress [3]. However, these studies principally focused on the relationship between serum iron concentration and thrombosis caused by oxidative stress, rarely mentioning whether a correlation existed between serum iron concentration and the components of thrombi due to oxidative stress. Additionally, we previously found that serum iron levels might be positively associated with the erythrocyte fraction in thrombi [4]. Thus, the present study, based on our previous study, is aimed at investigating whether the erythrocyte fraction in thrombi was increased with increasing serum iron concentration due to oxidative stress.

## 2. Materials and Methods

### 2.1. Patient Enrollment

Consecutive patients with acute ischemic stroke (AIS) who received endovascular treatment (EVT) in the Second Affiliated Hospital of Harbin Medical University from October to December 2019 were enrolled for this retrospective study. This study was approved by the ethics committee of the Second Affiliated Hospital of Harbin Medical University (KY2019-168) and conducted in accordance with the Declaration of Helsinki (1964). Each patient provided written consent. The authors had access to the information that could identify individual participants.

Patients in this study conformed to inclusion and exclusion criteria based on the latest guidelines on EVT for AIS [5]. Inclusion criteria are as follows: (1) AIS diagnosis, (2) large intracranial artery occlusions, (3) age ≥ 18 years, (4) premorbid modified Rankin scale (mRS) scores < 2, (5) National Institute of Health Stroke Scale (NIHSS) scores ≥ 6 and Alberta Stroke Program Early Computed Tomography Score (ASPECTS) ≥ 6, (6) MT within 6 h of stroke onset or treated 6–16 h based on DAWN or DEFUSE 3 eligibility criteria [6, 7], and (7) no findings on concomitant intracranial hemorrhagic disease, including aneurysm or arteriovenous malformations. The exclusion criteria are patients without retrieving thrombi. All patients were divided into two groups, namely, the erythrocyte-rich thrombi group and the fibrin-rich thrombi group, which was based on the erythrocyte fraction or fibrin fraction in thrombi that was more than 50%.

### 2.2. Endovascular Treatment Procedure

Before EVT, patients with AIS were diagnosed by neurologists based on their symptoms and by using medical imaging tools including computed tomography (CT) and magnetic resonance imaging (MRI). Moreover, physical examination and some other examinations including electrocardiography and blood tests were conducted. Based on the results, the neurointerventionists decided whether patients with AIS should receive EVT.

In accordance with the latest guidelines for AIS, patients received intravenous treatment (IVT) with recombinant tissue-type plasminogen activator (rt-PA) prior to EVT within 4.5 h of the presentation of symptoms. Otherwise, they received EVT directly, without any IVT, beyond 4.5 h. Thrombectomy was performed using the Solumbra technique [8], wherein a stent retriever was combined with a noncontact aspiration technique using intracranial catheter support. Lastly, modified thrombolysis in cerebral infarction (mTICI) 2b or 3 was deemed as successful recanalization. All thrombi were fixed in 10% neutralized buffered formalin immediately after retrieval.

### 2.3. Clinical Data Collection

Clinical data including baseline characteristics and blood tests were collected in this study. The baseline characteristics included age, sex (male), hypertension, diabetes mellitus (DM), atrial fibrillation (AF), history of ischemic stroke, smoking (defined as a patient who smoked ≥ 1 cigarette daily and continuously for six months), NIHSS by physical examination, and ASPECTS by CT or MRI. Further, the number of patients who received rt-PA prior to EVT, the number of patients treated by EVT beyond the time window, symptom onset to groin puncture time (OTP), sites of occlusion judged using CT angiography (CTA), or magnetic resonance angiography (MRA) and confirmed using digital subtraction angiography (DSA) including internal carotid artery (ICA), middle cerebral artery (MCA), and basilar artery (BA) and stroke causes judged by TOSAT classification were also included. Blood tests consisted of some possible items associated with thrombi, including prothrombin time (PT), prothrombin activity (PTA), activated partial thromboplastin time (APTT), fibrinogen (FIB), and thrombin time (TT).

### 2.4. Animals

Ten-week-old male Sprague-Dawley (SD) rats were purchased from the Animal Center of the Second Affiliated Hospital of Harbin Medical University. The animal study was approved by the animal ethical committee of the Second Affiliated Hospital of Harbin Medical University (SYDW2021-017) and conducted in accordance with the international guidelines regarding animal experimentation. All rats were kept in the animal laboratory of the Second Affiliated Hospital of Harbin Medical University, and all rats were fed with a specific pathogen-free class of rat diet and free access to water.

SD rats (*n* = 90) were randomly divided into the high-iron (HI) group (*n* = 12), medium-iron (MI) group (*n* = 12), low-iron (LI) group (*n* = 12), normal-saline (NS) group (*n* = 12), negative control (NC) group (*n* = 12), sham-operated (Sham) group (*n* = 6), iron group (*n* = 6), iron with DL-cysteine group (*n* = 6), control group (*n* = 6), and control with DL-cysteine group (*n* = 6). Iron-dextrin (Sigma-Aldrich, St. Louis, MO) was administered *in vivo* by intraperitoneal injection every other day for 5 weeks, for a total of 18 times. The body weights of rats was measured before the injection. Iron-dextrin was administered at a dose of 150 mg/kg in the HI group, 100 mg/kg in the MI, iron, and iron with DL-cysteine groups, or 50 mg/kg in the LI group based on body weight. Rats in the NS and sham groups received the same dosage of NS solution as that of iron-dextrin in the HI group, and the rats in the control and control with DL-cysteine groups received the same dosage of normal saline solution as that of iron-dextrin in the iron and iron with DL-cysteine groups. Rats in the NC group did not receive any injections. Next, 6 rats were randomly selected each from the HI, MI, LI, NS, and NC groups and the 6 rats in the sham group and 24 rats in the iron group, iron with DL-cysteine group, control group, and control with DL-cysteine group were used to establish the carotid artery thrombus model. Blood samples were collected after 5 weeks from the remaining rats. No significant differences in behavioral phenotypes were recorded between each group of rats during the treatment phases.

### 2.5. FeCl_3_-Induced Carotid Artery Thrombus Model

An FeCl_3_-induced carotid artery thrombus model was used to create thrombi in the carotid artery *in vivo* and examine their components, based on a previously published protocol with slight modifications [9]. Briefly, rats were anesthetized and the left carotid artery was isolated. A 5 × 5 mm piece of filter paper presoaked in 10% FeCl_3_ solution was wrapped around the adventitia of the left carotid artery for 30 min. In the sham group, the left carotid artery was isolated for 30 min, but without the insertion of the filter paper. Some rats in the MI and NS groups received an injection of DL-cysteine hydrochloride (Yuanye Biotechnology, Shanghai, China) in phosphate-buffered saline (PBS). Either DL-cysteine hydrochloride, a nonspecific ROS scavenger, at a dose of 50 mg/kg based on the body weight or the same dosage of PBS was administered via the tail vein 5 min after injury initiation. The left carotid artery was ligated and severed 2 mm above and below the filter paper after 30 min, after which the artery containing the thrombus was fixed in 10% neutralized buffered formalin and embedded in paraffin.

### 2.6. Blood Sample Collection

Clinical blood samples were collected from all enrolled patients within 2 h of EVT. Blood samples *in vivo* were collected from rats from the abdominal aorta following general anesthesia. All blood samples were collected in EDTA-containing anticoagulant tubes and immediately centrifuged, aliquoted, and stored at −80°C until analysis. Clinical blood samples were stored within 6 months, as well as blood samples *in vivo* within 1 week.

### 2.7. Hematoxylin and Eosin (HE) Staining

HE staining (Solarbio, Beijing, China) was performed following previously described standard procedures [10]. Briefly, sections containing the retrieved thrombi and left carotid arteries with the thrombi were stained with HE at room temperature for 1 min. Hematoxylin stains the basophilic components while eosin stains acidophilic components. Images of HE sections were acquired at 100x or 200x magnification (sham group only at 100x magnification), captured using an Eclipse TI microscope (Nikon, Tokyo, Japan), and stored in a digital format. ImageJ (National Institutes of Health, Bethesda, MD, USA) was used to measure the area proportion consisting of red blood cells or fibrin (100x magnification), which represented the erythrocyte or fibrin fractions in thrombi. Histopathological analysis was conducted by investigators blinded to clinical and animal data.

### 2.8. Immunofluorescence Staining

Immunofluorescence staining was performed to examine the red blood cells and fibrin (OGEN) in thrombi following a previously reported procedure [11]. Briefly, antigen retrieval was performed on sections containing the left carotid artery and thrombi by heating to 95°C for 20 min in sodium citrate buffer (pH 6). The sections were then washed three times for 5 min with PBS, blocked in 10% normal sheep serum for 30 min, and incubated overnight at 4°C with fibrinogen polyclonal antibody (1 : 300; Thermo Fisher Scientific, Waltham, USA) in PBS. The sections were washed in PBS three times for 5 min after warming to room temperature for 30 min. Lastly, the sections were incubated with Alexa Fluor 488 donkey anti-sheep IgG (1 : 500; Abcam, Cambridge, UK) in PBS at room temperature for 60 min and washed three times for 5 min. Negative controls of immunofluorescence staining were achieved by omission of the primary antibody and by using the isotype primary antibody (Supplemental Figure (available here)). Omission controls and isotype controls were performed by incubating a section with the diluent of the primary antibody (PBS) and the isotype primary antibody in PBS (sheep IgG isotype control antibody (Thermo Fisher Scientific, Waltham, USA)). The red blood cells were visualized at a wavelength of 555 nm based on their inherent fluorescence. Images were acquired at 400x magnification using an Eclipse TI inverted fluorescence microscope and processed using NIS-Elements F software (Nikon, Tokyo, Japan). ImageJ was used to analyze the relative tightness of fibrin networks at 400x magnification represented by the average of fibrin fibers per field (1 *μ*m^2^) in the fibrin networks. The average was calculated from the number of fibrin fibers from three different fields in the fibrin networks. Histopathological analysis was conducted by investigators blinded to the animal data.

### 2.9. Analysis of Blood Samples

Serum iron levels in the enrolled patients were determined using colorimetry with 2,2-dipyridine-bipyridine (Solarbio, Beijing, China) with a previously described method [12]. Serum iron levels in rats were measured colorimetrically using an assay kit (Jiancheng Bioengineering Institute, Nanjing, China). Plasma levels of lipid oxidation products, including malondialdehyde (MDA), and antioxidative enzymes including superoxide dismutase (SOD), catalase (CAT), and glutathione peroxidase (GSH-PX) were measured using the corresponding enzyme-linked immunosorbent assay (ELISA) kits (Meimian, Jiangsu, China). The increase in lipid oxidation products and decrease in antioxidative enzyme activity indicated increasing oxidative stress levels.

### 2.10. Preparation of Buffers and Fibrin Clot Formation *In Vitro*

Both the diluting and percolating buffers were 0.05 M Tris-HCl and 0.15 M NaCl (pH 7.5; adjusted with HCl). Human fibrinogen lyophilized powder (Sigma-Aldrich, St. Louis, MO) was dissolved in the diluting buffer. The fibrin clot was mixed at room temperature for 1 h such that the final concentration of the solution was 2 mg/L human fibrinogen, 20 mM CaCl_2_, and 1 U/mL human thrombin (Sigma-Aldrich, St. Louis, MO). Different concentrations of H_2_O_2_ were added before fibrin clot formation, which resulted in the final concentrations of 0.9, 0.6, 0.3, and 0 mM H_2_O_2_ (for 0 mM, H_2_O_2_ was replaced with the same volume of deionized water).

### 2.11. Fibrin Clot Permeability

Fibrin clot permeability was measured to assess the tightness of fibrin networks. Permeability was evaluated using a modified pressure-driven system in accordance with a previously described method [13]. Briefly, a modified flat-bottomed centrifuge tube containing the fibrin clot was connected to the outer cup of a syringe containing percolation buffer. The percolation buffer (10 mL) was used to wash away any soluble liquid in the flat-bottomed tube after removing air from the system, and the time required for 50 mL of the percolation buffer to flow through the fibrin clot was recorded. The permeation coefficient (Ks) was calculated as follows: Ks = *Q* × *Lη*/*t* × *A* × Δ*p*, where *Q* (mL) represents the flow rate in time *t* (sec), *L* is the length of the fibrin clot (cm), *η* is the viscosity of the liquid (g/cm^∗^s), *A* is the cross-sectional area (cm^2^), and Δ*p* is the differential pressure (dyne/cm^2^). The experiment was performed three times for each group.

### 2.12. Statistical Analysis

Data are presented as mean ± standard deviation (SD) or mean ± standard error of the mean (SEM). Unpaired Student's *t*-test or Mann-Whitney *U* test was used to examine the differences between two groups after checking the normality of distribution using the Kolmogorov-Smirnov test or a one-way ANOVA for multiple groups with post hoc contrasts by the Student-Newman-Keuls test, as appropriate. Linear regression was used to establish the relationship between two groups of continuous variables. GraphPad Prism (version 8.0) and SPSS (version 22.0) were used for statistical analysis. At least three independent experiments *in vitro* were performed in each group, for which *n* represents the number of rats in each group or the number of experiment repeats. *p* < 0.05 was considered statistically significant.

## 3. Results

### 3.1. Correlation of Serum Iron in the Erythrocyte Fraction in the Thrombi from Clinical Samples

A total of 30 patients with AIS including 17 males and 13 females were enrolled from 37 patients with AIS who underwent EVT between October and December 2019. None of the patients had a history of coagulation abnormalities or iron imbalance-related disease. For EVT, a Solitaire FR (Medtronic, USA) was used for thrombectomy.

**Figure 1:**
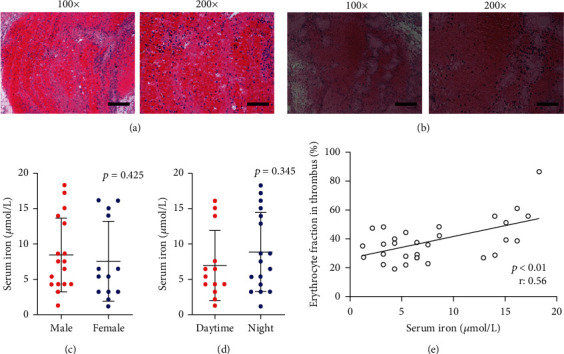
Histopathologic analysis of the retrieved thrombi and clinical outcomes on the correlation between serum iron and erythrocyte fraction in thrombi. HE images of the retrieved thrombi: red blood cells are in red and granulating area and fibrin are in pink. (a) Retrieved thrombi with relatively high erythrocyte fraction in thrombi. (b) Retrieved thrombi with relatively low erythrocyte fraction in thrombi. (c) Serum iron level in male patients (8.44 ± 1.26 *μ*mol/L) was not significantly higher than that in female patients (7.55 ± 1.57 *μ*mol/L) (*p* = 0.425). (d) Serum iron levels of blood samples collected at daytime (6.98 ± 1.37 *μ*mol/L) were not significantly higher than those of samples collected at night (8.88 ± 1.36 *μ*mol/L) (*p* = 0.345). (e) Serum iron was positively associated with erythrocyte fraction in thrombi (*p* < 0.01, *r*: 0.56). Results are expressed as mean ± SEM. Scale bar = 200 *μ*m at 100x magnification or 100 *μ*m at 200x magnification.

To determine the relationship between erythrocyte fraction in thrombi and serum iron, based on our previous study [4], HE images of the retrieved thrombi were used to measure and analyze the erythrocyte fractions (Figures 1(a) and 1(b)). The basic characteristics of all patients are listed in Table 1, and no significant differences were found between patients in the erythrocyte-rich thrombi group and fibrin-rich thrombi group. Only the serum iron levels of patients in the erythrocyte-rich thrombi group were significantly higher than those in the fibrin-rich thrombi group instead of PT, PTA, APTT, FIB, and TT, indicating that serum iron might be associated with erythrocyte-rich thrombi (Table 2). Because serum iron concentration might be influenced by differences in gender and time of blood collection as reported in previous studies [14, 15], we categorized the enrolled patients into subgroups based on gender (male or female) and time of blood collection (daytime: 8 : 00 to 15 : 00 or night: 15 : 00 to 8 : 00) to further analyze the possible interference factors. No significant differences were apparent between the gender subgroups (Figure 1(c)) and blood collection time subgroups (Figure 1(d)), suggesting that serum iron concentration was not influenced by gender or the time of blood collection in this study. We further explored the relationship between serum iron and erythrocyte fraction in thrombi, and the results from the linear regression analysis indicated a positive association (Figure 1(e)). However, it remained unclear about the causal relationship between serum iron concentration and erythrocyte fraction in thrombi.

### 3.2. Correlation between Serum Iron Levels and Erythrocyte Fraction in Thrombi In Vivo

**Figure 2:**
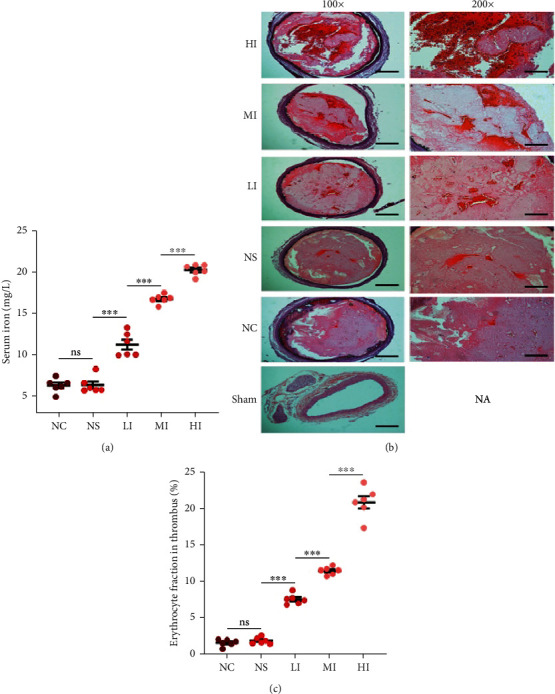
Correlation of serum iron and erythrocyte fraction in thrombi *in vivo*. (a) There were significant differences between the HI (20.26 ± 0.26 *μ*mol/L), MI (16.75 ± 0.22 *μ*mol/L), and LI groups (11.27 ± 1.43 *μ*mol/L) when compared with the NC group (6.37 ± 0.82 *μ*mol/L), but the not in the NS group (6.42 ± 0.41 *μ*mol/L) (*p* = 0.923). Serum iron concentration was increased with the administration of iron-dextrin. (b) HE images of rat carotid arteries with the thrombi in each group. Red blood cells are in red and granulating area and fibrin are in pink. There were no thrombi in the carotid arteries of rats in the sham group. (c) Significant differences in erythrocyte fraction in the thrombi between the HI (20.80 ± 0.84%), MI (11.43 ± 0.53%), LI (7.55 ± 0.70%), and NC groups (1.63 ± 0.47%) and no significant differences between the NS (1.92 ± 0.42%) and NC groups (*p* = 0.642). Erythrocyte fraction in thrombi increased in the presence of serum iron. Results are expressed as mean ± SEM, *n* = 6; ns: *p* > 0.05; ^∗∗∗^*p* < 0.001. Scale bar = 200 *μ*m at 100x magnification or 100 *μ*m at 200x magnification.

Before further investigating the correlation between serum iron levels and erythrocyte fraction in thrombi, serum iron concentrations should be determined after receiving the injection *in vivo* [16]. The serum iron levels in the HI, MI, and LI groups were significantly different than those in the NC group and increased with an increase in iron-dextrin, but there were no significant differences between the NS and NC groups (Figure 2(a)). These results suggested that serum iron levels increased significantly with an increase in the dosage of iron-dextrin.

The thrombi produced *in vivo* in the carotid arteries in all groups except the sham group were stained using HE (Figure 2(b)), which demonstrated that surgery to isolate the left carotid artery did not result in thrombosis [17]. Significant differences in the erythrocyte fraction were found in all groups except for the NS group, in comparison with the NC group. The highest erythrocyte fraction in the thrombi was found in the HI group, and a significant decrease was found in the MI and LI groups (Figure 2(c)). These results indicated that the erythrocyte fraction in thrombi increased with serum iron, thereby establishing the primary and secondary relationships between serum iron and erythrocyte fraction in thrombi.

**Figure 3:**
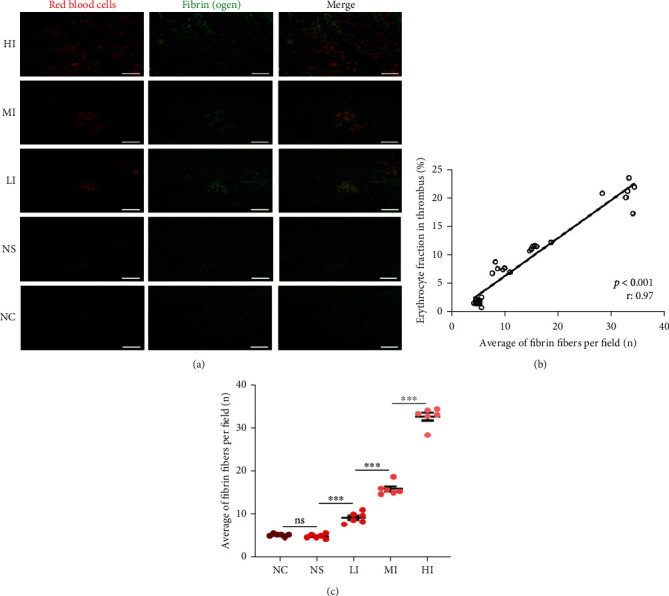
Correlation of serum iron and the tightness of fibrin networks *in vivo*. (a) Immunofluorescence images of rat carotid arteries with the thrombi in each group show fibrin networks in a net-shaped area. (b) Erythrocyte fraction in thrombi was positively associated with the average number of fibrin fibers per field (*p* < 0.001, *r*: 0.97). (c) Significant differences in the average number of fibrin fibers per field between the HI (32.60 ± 0.89), MI (15.90 ± 0.59), LI (9.23 ± 0.50), and NC groups (5.22 ± 0.34) and no significant differences between the NS (4.95 ± 0.20) and NC groups (*p* = 0.730). The average number of fibrin fibers per field increased in the presence of serum iron. Results are expressed as mean ± SEM, *n* = 6; ns: *p* > 0.05; ^∗∗∗^*p* < 0.001. Scale bar = 50 *μ*m at 400x magnification.

Previous studies have reported that red blood cells are captured by the fibrin network during thrombus formation [18, 19]. Thus, it could be deduced that the erythrocyte fraction in thrombi was regulated by the tightness of the fibrin networks. Immunofluorescence studies showed that red blood cells in the thrombi were scattered and embedded within the fibrin network (Figure 3(a)). Based on the immunofluorescence images, we also confirmed that the erythrocyte fraction in thrombi was positively associated with the average number of fibrin fibers per field (Figure 3(b)). Furthermore, there were significant differences in the average number of fibrin fibers per field in the HI, MI, and LI groups compared with those in the NC group. No significant differences were found between the NS and NC groups with respect to fibrin fibers. In addition, the average number of fibrin fibers per field increased with an increase in serum iron levels (Figure 3(c)). These results suggested that the erythrocyte fraction in thrombi was increased with an increase in serum iron and was attributed to the regulation of the tightness of the fibrin network.

### 3.3. Role of Oxidative Stress on the Correlation between Serum Iron and Erythrocyte Fraction in Thrombi In Vivo and In Vitro

**Figure 4:**
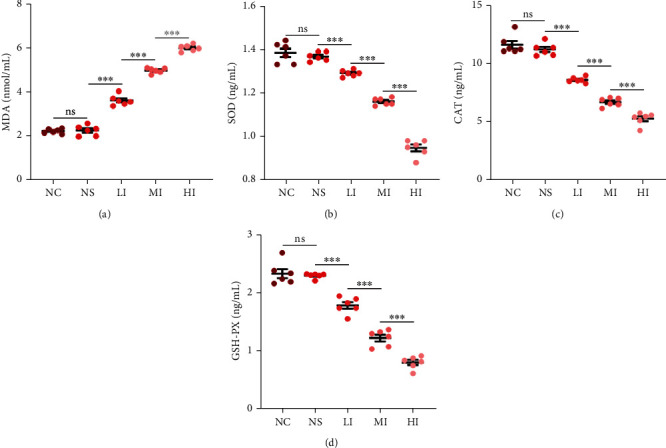
Correlation of serum iron and oxidative stress level *in vivo*. (a) Significant differences in MDA concentration between the HI (5.99 ± 0.06 nmol/L), MI (4.97 ± 0.05 nmol/L), LI (3.63 ± 0.09 nmol/L), and NC groups (2.25 ± 0.10 nmol/L) and no significant differences between the NS (2.27 ± 0.09 nmol/L) and NC groups (*p* = 0.779). MDA concentration was increased in the presence of serum iron. (b) Significant differences in SOD concentration between the HI (0.95 ± 0.02 ng/mL), MI, (1.16 ± 0.01 ng/mL), LI (1.29 ± 0.01 ng/mL), and NC groups (1.38 ± 0.05 ng/mL) and no significant differences between the NS (1.37 ± 0.01 ng/mL) and NC groups (*p* = 0.346). SOD concentration decreased in the presence of serum iron. (c) Significant differences in CAT concentration between the HI (5.30 ± 0.21 ng/mL), MI (6.73 ± 0.14 ng/mL), LI (8.64 ± 0.09 ng/mL), and NC groups (11.63 ± 0.33 ng/mL) and no significant differences between the NS (11.23 ± 0.22 ng/mL) and NC groups (*p* = 0.206). CAT concentration decreased in the presence of serum iron. (d) Significant differences in GSH-PX concentration between the HI (0.80 ± 0.04 ng/mL), MI (1.21 ± 0.06 ng/mL), LI (1.78 ± 0.06 ng/mL), and NC groups (2.33 ± 0.08 ng/mL) and no significant differences between the NS (2.29 ± 0.02 ng/mL) and NC groups (*p* = 0.639). GSH-PX concentration was decreased in the presence of serum iron. Results are expressed as mean ± SEM, *n* = 6; ns: *p* > 0.05; ^∗∗∗^*p* < 0.001.

It was certain that investigating the influence of oxidative stress on the correlation of serum iron and erythrocyte fraction was to investigate whether oxidative stress could influence the relationship between serum iron and the tightness of fibrin networks. To examine the relationship between oxidative stress and serum iron levels, MDA, SOD, CAT, and GSH-PX levels in all groups were analyzed [20, 21]. MDA levels in the HI, MI, and LI groups were significantly different from those in the NC group, whereas there were no significant differences in MDA levels between the NS and NC groups. Additionally, the increase in MDA levels was significantly associated with increased serum iron levels (Figure 4(a)). Meanwhile, SOD, CAT, and GSH-PX levels in all groups, except the NS group, were significantly different compared with those in the NC group and revealed a significantly negative association between serum iron levels and SOD levels (Figures 4(b)–4(d)). The results suggested that oxidative stress was enhanced with an increase in serum iron levels.

**Figure 5:**
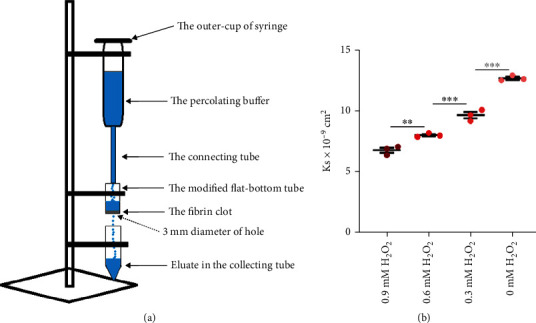
Correlation of the oxidative stress level and tightness of fibrin networks *in vitro*. (a) Components of the modified pressure-driven system and the principle of measuring fibrin clot permeability. (b) Ks values of fibrin clots in 0.9 mM H_2_O_2_ (6.80 ± 0.81^∗^10^−9^ cm^2^) were significantly lower than those of fibrin clots in 0.6 mM H_2_O_2_. Ks values of fibrin clots in 0.6 mM H_2_O_2_ (8.03 ± 0.09^∗^10^−9^ cm^2^) were significantly lower than those of fibrin clots in 0.3 mM H_2_O_2_. Ks values of fibrin clots in 0.3 mM H_2_O_2_ (9.67 ± 0.26^∗^10^−9^ cm^2^) were significantly lower than those of fibrin clots in 0 mM H_2_O_2_ (12.67 ± 0.12^∗^10^−9^ cm^2^). Ks values decreased significantly with increasing H_2_O_2_ concentration. Results are expressed as mean ± SEM, *n* = 3; ^∗∗^*p* < 0.01; ^∗∗∗^*p* < 0.001.

The Ks value was calculated to determine the pore size within the fibrin networks, wherein a smaller pore size represented a tighter fibrin network [22]. Furthermore, H_2_O_2_ was added to the fibrin clots to simulate the influence of oxidative stress on the fibrin network formation. The higher the H_2_O_2_ concentration, the higher was the oxidative stress [23]. A modified pressure-driven system to evaluate the permeability of fibrin clots is shown in Figure 5(a). We found a significant difference in Ks values of the fibrin clots with different H_2_O_2_ concentrations. Fibrin clots treated with higher H_2_O_2_ concentrations were determined to have lower Ks values (Figure 5(b)). The smaller pore size in the fibrin networks in the fibrin clots resulting from higher H_2_O_2_ concentrations revealed that a tighter fibrin network was formed as the H_2_O_2_ concentration was increased. These results indicated that an increase in oxidative stress, as found with higher levels of serum iron, resulted in the formation of tighter fibrin networks.

**Figure 6:**
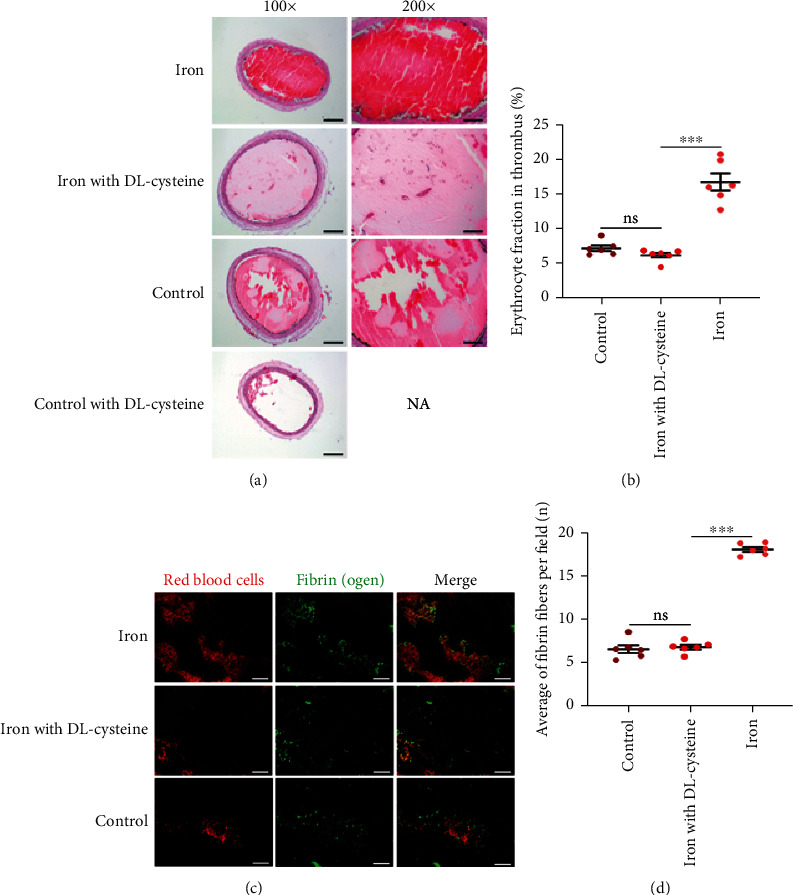
Correlation of serum iron and erythrocyte fraction in thrombi or the tightness of fibrin networks after inhibiting oxidative stress *in vivo*. (a) HE images of rat carotid arteries with the thrombi in each group. There were no thrombi in the control with DL-cysteine group. (b) Erythrocyte fraction in thrombi in the iron group (16.58 ± 1.24%) was significantly higher than that in the iron with DL-cysteine group (6.12 ± 0.36%) and control group (7.12 ± 0.40%). There were no significant differences in erythrocyte fraction in thrombi between the iron with DL-cysteine group and control group (*p* = 0.379). (c) Immunofluorescence images of rat carotid arteries with the thrombi in the iron group, iron with DL-cysteine group, and control group. (d) Fibrin networks in the iron group (18.00 ± 0.28) were significantly tighter than those in the iron with DL-cysteine group (6.82 ± 0.27) and control group (6.58 ± 0.45). There were no significant differences in the tightness of fibrin networks between the iron with DL-cysteine group and control group (*p* = 0.635). Results are expressed as mean ± SEM, *n* = 6; ns: *p* > 0.05; ^∗∗∗^*p* < 0.001.

To further determine the role of oxidative stress, experiments *in vivo* were performed wherein oxidative stress was inhibited using DL-cysteine hydrochloride [24]. HE images showed no obvious thrombi were found in the severed carotid arteries in control with DL-cysteine group, and it might be attributed to DL-cysteine which influenced FeCl_3_-induced carotid artery thrombus model by inhibiting oxidative stress (Figure 6(a)). Findings from HE staining revealed that the erythrocyte fraction in thrombi in the iron group was significantly higher than that in the iron with DL-cysteine group and the control group, and that there were no significant differences between the iron with DL-cysteine group and the control group (Figure 6(b)). Immunofluorescence images showed fibrin networks in the iron group, iron with DL-cysteine group, and the control group (Figure 6(c)). The fibrin networks in the iron group were significantly tighter than those in the iron with DL-cysteine group and the control group, whereas the tightness of fibrin networks in the iron with DL-cysteine group and the control group was not significantly different (Figure 6(d)). These results suggested that inhibiting oxidative stress during iron overload may reduce the erythrocyte fraction and the tightness of fibrin networks in thrombi, which further indicated that the involvement of oxidative stress led to an increase in the erythrocyte fraction in thrombi in the presence of serum iron and could influence the fibrin networks.

## 4. Discussion

In this study, we investigated the relationship between the erythrocyte fraction in thrombi and serum iron from patients and also studied this aspect using experiments *in vivo* and *in vitro*. As red blood cells in thrombi were captured by the fibrin networks, we further explored the relationship between fibrin networks and serum iron. Although previous studies have reported that iron can lead to the formation of denser fibrin networks *in vitro* [25, 26], these studies utilized inorganic iron ions to influence the fibrin networks, which are likely in a different form than those in the body. Additionally, the published studies have not investigated whether oxidative stress is involved in the influence of iron on fibrin networks. Therefore, in this study, we used serum iron, which is similar to iron in the body, as a marker to investigate our hypothesis.

Under physiological conditions, various proteins react with iron to generate significant quantities of ROS through biological processes [27]. A previous study has established that iron overload increases local ROS generation in vessel walls via the production of both OH^•^ and H_2_O_2_ at the site of thrombi [3]. It was affirmed that iron played a role leading to oxidative stress in the body. Nevertheless, it was speculated in the study that ROS might be generated in blood and could directly influence fibrin networks as they were being formed. This speculation was explained by previous findings that iron induces neutrophils in the blood to release ROS or that iron-mediated Fenton reactions could increase ROS production [28, 29]. It should be also noted that thrombi were not found in the control with DL-cysteine group. This phenomenon could be explained based on the previous study that oxidative stress leads to thrombosis in a model of FeCl_3_-induced carotid artery thrombus [30] but DL-cysteine inhibits oxidative stress and prevents thrombus formation in the control with DL-cysteine group. However, the involvement of oxidative stress could be still proven by results from the other groups based on experiments *in vivo* with the inhibition of oxidative stress, because a similar thrombus model was used in our study.

Previous studies have reported that the production of two-stranded protofibrils and the formation of fibrin networks are involved in the fibrin polymerization process [31, 32]. Removal of the central region in fibrinopeptides and the intrinsic flexible region of the *α*C connector facilitates the transition from intramolecular to intermolecular interactions [33]. The crystal packing of fragment D and its covalently bound dimer might be promoted through end-to-end associations [34]. Monomeric molecules can be connected through D-E-D interactions to form two-stranded protofibrils and the extended *γ*-chain in the crosslinks are present externally after forming the two-stranded protofibrils [35]. First, the *α*C-*α*C domains are untethered in the lateral connections of the protofibrils. Subsequently, the untethered *α*C domains from one protofibril interact with the another *α*C domains [36]. As the two-stranded protofibrils propagate longitudinally and reach a certain length, they aggregate laterally to form thicker fibers or multiple strands of fibers that combine to form twisted fibrin fibers and bundles and then branch off at several points to form interwoven fibrin networks [37, 38]. However, a lack of interaction sites in the *α*C domain of fibrinogen due to oxidative stress is considered the principal cause for impaired lateral association [39]. Dissociation of the *α*C domain increases intermolecular binding and enhances the interaction between the mutually aligned *γ*-chains [40]. Additionally, the end-linked dimers might be able to alter the conformations of the D and DD regions during fibrin assembly and lead to the formation of highly branched thinner fibers as opposed to the normal fibrin architecture that is observed due to the two-dimensional stacking behavior influenced by the primary orientation of fibrinogen [41]. Findings from atomic force microscopy from a previous study suggest that oxidative stress influences the connections of protofibrils and the crosslinking patterns between the *α*- and *γ*-chains, resulting in more branching and denser aggregation [42].

## 5. Limitations

There were some limitations in this study. First, statistical bias may have been present owing to the availability of limited samples from a single stroke center. However, our previous study that had enrolled more patients revealed similar findings and the current study further strengthens our previous findings. Although Ks values were used in this study to estimate the tightness of fibrin networks after H_2_O_2_ modifications in experiments *in vitro*, we did not obtain direct images using scanning electron microscopy. This would be needed in our further study. Lastly, we were only able to investigate the oxidative stress involved in the biological process and did not examine other factors such as ROS generation in blood or the possible signaling pathways. These mechanisms will be studied in our future studies.

## 6. Conclusions

Our findings demonstrated that oxidative stress was involved in the biological process that erythrocyte fraction in thrombi was increased with serum iron by influencing fibrin network formation.

## Figures and Tables

**Table 1:** Analysis of baseline characteristic in all enrolled patients.

	All patients (*n* = 30)	Erythrocyte-rich thrombi (*n* = 5)	Fibrin-rich thrombi (*n* = 25)	*p* value
Age, mean ± SD	63.2 ± 1.8	59.6 ± 1.7	63.9 ± 2.2	0.391
Male sex, *n* (%)	17 (56.7%)	3 (60.0%)	14 (56.0%)	>0.999
Smoking, *n* (%)	15 (50.0%)	3 (60.0%)	12 (48.0%)	>0.999
Hypertension, *n* (%)	13 (43.3%)	1 (20.0%)	12 (48.0%)	0.510
DM, *n* (%)	8 (26.6%)	2 (40.0%)	6 (24.0%)	0.854
AF, *n* (%)	10 (33.3%)	1 (20.0%)	9 (36.0%)	0.862
History of ischemic stroke, *n* (%)	6 (20.0%)	1 (20.0%)	5 (20.0%)	>0.999
NIHSS, mean ± SD	14.6 ± 0.5	15.8 ± 0.9	14.4 ± 0.6	0.238
ASPECTS, mean ± SD	8.7 ± 0.2	9.2 ± 0.4	8.6 ± 0.3	0.373
IVT, *n* (%)	3 (10.0%)	1 (20.0%)	2 (8.0%)	0.453
EVT beyond time window, *n* (%)	6 (20.0%)	1 (20.0%)	5 (20.0%)	>0.999
OTP (min)	294.8 ± 27.7	244.4 ± 44.5	304.9 ± 32.0	0.344
*Occlusion sites,n(%)*				0.711
ICA	9 (30.0%)	1 (20.0%)	8 (32.0%)	
MCA	13 (43.3%)	3 (60.0%)	10 (40.0%)	
BA	8 (26.7%)	1 (20.0%)	7 (28.0%)	
*Stroke cause,n(%)*				0.494
LAA	14 (46.7%)	2 (40.0%)	12 (48.0%)	
CE	10 (33.3%)	1 (20.0%)	9 (36.0%)	
Undetermined and others	6 (20.0%)	2 (40.0%)	4 (16.0%)	

DM: diabetes mellitus; AF: atrial fibrillation; NIHSS: National Institutes of Health Stroke Scale; ASPECTS: Alberta Stroke Program Early Computed Tomography Score; IVT: intravenous treatment; EVT: endovascular treatment; OTP: symptom onset to groin puncture time; ICA: internal carotid artery; MCA: middle cerebral artery; BA: basilar artery; LAA: large artery arteriosclerosis; CE: cardiogenic embolism.

**Table 2:** Analysis of the factors in blood test on erythrocyte-rich thrombi.

	Erythrocyte-rich thrombi (*n* = 5)	Fibrin-rich thrombi (*n* = 25)	*p* value
Serum iron (*μ*mol/L), mean ± SD	16.2 ± 0.8	6.4 ± 0.8	**0.001**
PT (sec), mean ± SD	11.2 ± 0.4	11.2 ± 0.2	0.982
PTA, mean ± SD	95.0 ± 6.7	96.8 ± 3.4	0.826
APTT (sec), mean ± SD	35.1 ± 2.8	33.0 ± 1.1	0.446
FIB (g/L), mean ± SD	2.9 ± 0.2	2.9 ± 0.1	0.813
TT (sec), mean ± SD	13.9 ± 0.8	15.4 ± 0.9	0.290

PT: prothrombin time; PTA: prothrombin activity; APTT: activated partial thromboplastin time; FIB: fibrinogen; TT: thrombin time.

## Data Availability

The datasets used and/or analyzed during the current study are available from the corresponding author upon reasonable request.

## Supplementary Materials

Supplementary Materials Supplemental Figure: omission of the primary antibody and the isotype primary antibody for negative controls of immunofluorescence staining.
