# Supplementary material for: Erythrocyte Fraction in Thrombi Is Increased with Serum Iron by Influencing Fibrin Networks via Oxidative Stress
Source: Oxid Med Cell Longev. 2021 Dec 24;2021:3673313. doi: 10.1155/2021/3673313 (PMC8719990; doi:10.1155/2021/3673313)

**Supplemental Figure Legends:**

**Supplemental Figure: Omission of the primary antibody and the isotype primary antibody for negative controls of immunofluorescence staining**

(A, B) Omission of the primary antibody and the isotype primary antibody for negative controls of immunofluorescence staining showed that no positive signal was detected for fibrin (green) but showed the presence of autofluorescence from red blood cells (red). Scale bar=50 μm at 400× magnification.

**Supplemental Figure:**


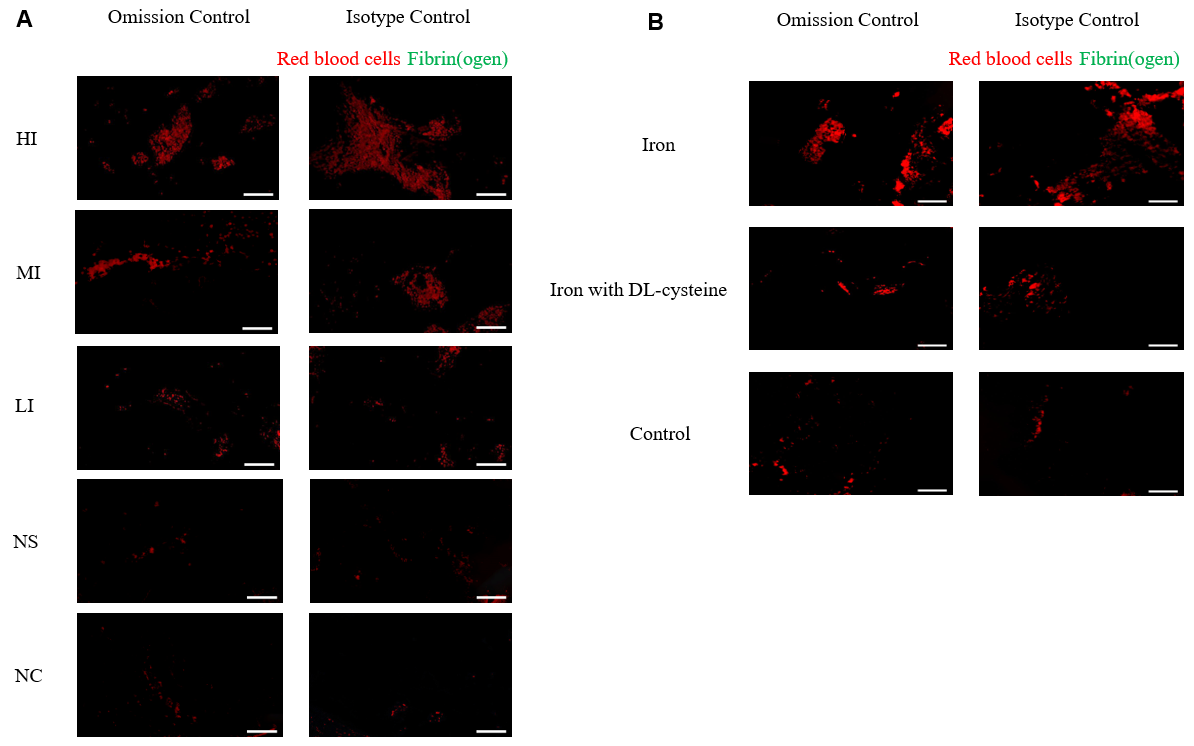

Supplement: Supplementary Materials — Supplemental Figure: omission of the primary antibody and the isotype primary antibody for negative controls of immunofluorescence staining. [file 3673313.f1.docx]
